# First person – Xiao-Zhi Cheng

**DOI:** 10.1242/bio.043752

**Published:** 2019-05-15

## Abstract

First Person is a series of interviews with the first authors of a selection of papers published in Biology Open, helping early-career researchers promote themselves alongside their papers. Xiao-Zhi Cheng is first author on ‘ Intercellular transfer of P-glycoprotein mediates the formation of stable multidrug resistance in human bladder cancer BIU-87 cells’, published in BiO. Xiao-Zhi conducted the research described in this article while a Master’s student in Professor Hui-Liang Zhou’s lab at Fujian Medical University, People's Republic of China. He is now an attending physician in the lab of Hui-liang Zhou at Huanggang Central Hospital, China, investigating the mechanism of multidrug resistance formation and the role of P-glycoprotein within it.


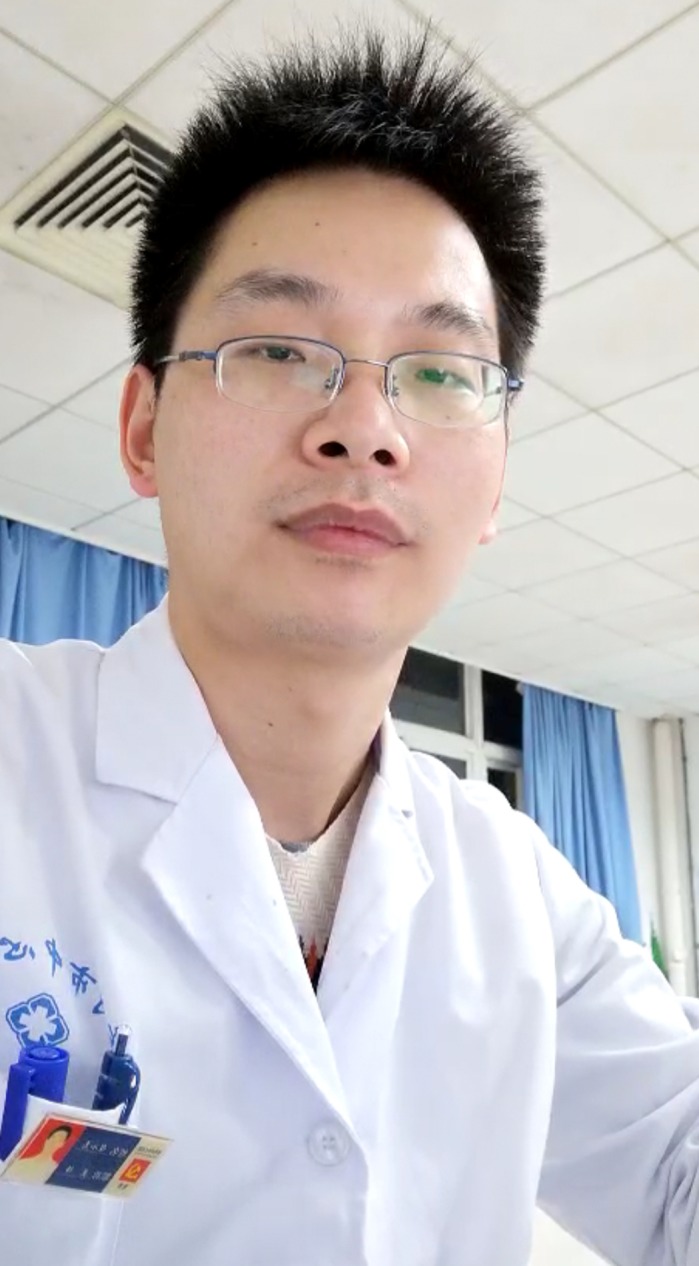


**Xiao-Zhi Cheng**

**What is your scientific background and the general focus of your lab?**

My bachelor's degree is clinical medicine and my master's degree is surgery. My main research interest is the mechanism by which cancer patients produce multidrug resistance during chemotherapy. The laboratory always focuses on the formation mechanism of multidrug resistance, especially the role of intercellular transfer of P-glycoprotein (P-gp).

**How would you explain the main findings of your paper to non-scientific family and friends?**

The formation of multidrug resistance is a complex process during chemotherapy in cancer patients. Only by finding the cause of multidrug resistance can we cure it. P-gp is an energy-dependent efflux pump on the surface of the cell membrane that pumps substances out of cells and causes multidrug resistance. Under the action of anti-tumor drugs, our study found that drug-resistant cells transfer P-gp to sensitive cells, making sensitive cells less susceptible to drug effects, and ultimately forming drug resistance, enhancing the overall drug resistance of the tumor. This is a new mechanism for the formation of multidrug resistance.

“Only by finding the cause of multidrug resistance can we cure it.”

**What are the potential implications of these results for your field of research?**

Intercellular transfer of P-gp is a new mechanism of multidrug resistance. Blocking the transfer of P-gp between cells can increase the sensitivity of chemotherapy. Especially when it comes to ineffective chemotherapy, this mechanism should be considered.

**What has surprised you the most while conducting your research?**

We initially thought that the transfer of P-gp levels would disrupt the cell's long-lasting multidrug resistance (MDR), as studies found that acquired drug-resistant cells (AqMDRs) died gradually in medium containing 1 μg/ml doxorubicin (DOX). However, this is not the case. There was no significant decrease in the P-gp content of AqMDRs cells in DOX-containing medium. Further studies of MDR1 mRNA indicate that the expression level of MDR1 mRNA gradually increased. The cells themselves upregulate MDR1 mRNA expression, maintain MDR characteristics and survive.

**What, in your opinion, are some of the greatest achievements in your field and how has this influenced your research?**

Our previous studies indirectly demonstrated that microparticles (MPs) were involved in intercellular P-gp transfer. It has been reported that MPs exhibit tissue selectivity in the process of transferring drug-resistant proteins and carrying nucleic acids. Next, biological characteristics of MPs will be further explored. If we can assemble targeted drugs into MPs, it may be important in the treatment of tumors.

**Figure:**
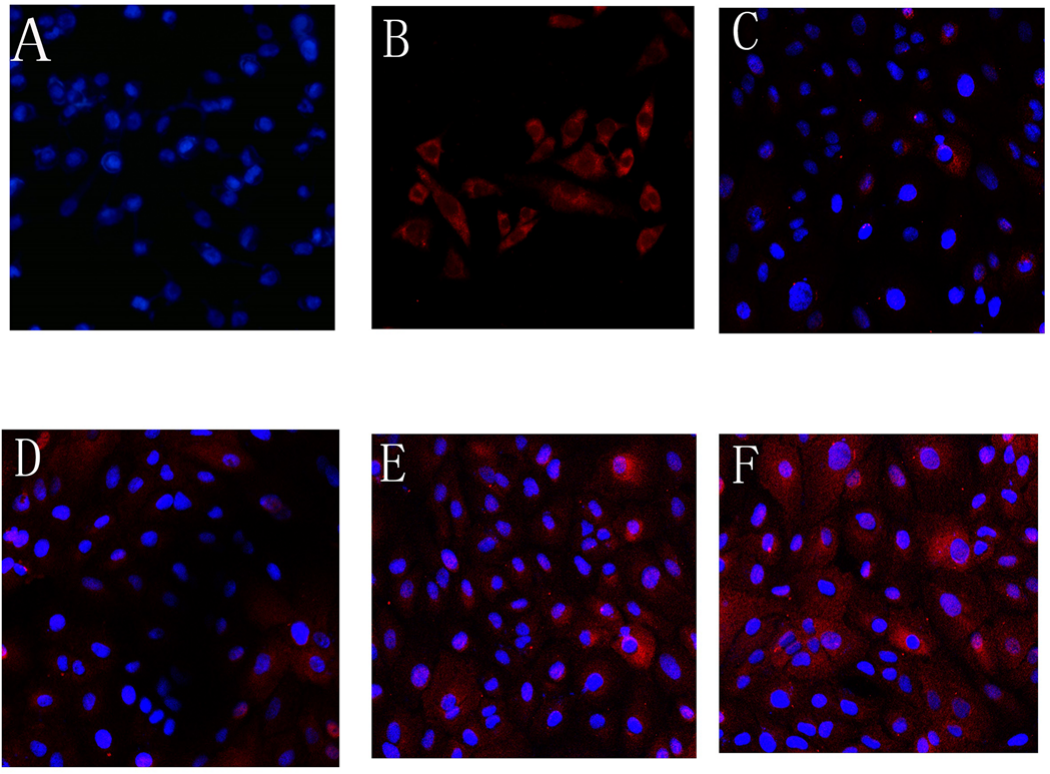
**Multidrug-resistant tumors are not terrible, if terrible means that we don't know anything about them.**

**What changes do you think could improve the professional lives of early-career scientists?**

At first, young scientists should read literature extensively and become familiar with the latest findings in the field. Reading and trying to write review articles is a good training tool. Secondly, it is necessary to carry out more experiments and propose innovative experimental methods based on the proficiency of experimental methods in this field. Finally, we must think more, be good at finding problems, solve problems and continue to grow in practice.

**What's next for you?**

Next, I'm going to pursue a doctorate. At the same time – as I found that the MPs were magical particles – I will continue to study and find more meaningful discoveries for cancer patients.
